# The earliest Mesopsychidae and revision of the family Mesopanorpodidae (Mecoptera)

**DOI:** 10.3897/zookeys.130.1611

**Published:** 2011-09-24

**Authors:** Alexei S. Bashkuev

**Affiliations:** 1 Borissiak Paleontological Institute, Russian Academy of Sciences, Moscow, 117997, Russia

**Keywords:** *Mesopsychidae*, *Mesopanorpodes*, *Mesopsyche*, *Permopsyche*, *Bittacopanorpa*, Permian, Triassic, new taxa, revision

## Abstract

The family Mesopanorpodidae is revised. Most taxa referred to this family are not related to the type genus *Mesopanorpodes* Tillyard, 1918. The type species of the latter, *Mesopanorpodes wianamattensis*,is closely related to *Mesopsyche* Tillyard, 1917. Therefore *Mesopanorpodes* is transferred to Mesopsychidae Tillyard, 1917 (= Mesopanorpodidae Tillyard, 1918, **syn. n.**).The earliest Mesopsychidae are described from the Upper Permian of European Russia (Severodvinian; Isady locality, Vologda Province): *Permopsyche issadensis* **gen. etsp. n.** (type species) and *Permopsyche rasnitsyni* **sp. n.** Two species described under *Mesopanorpodes* from the Upper Permian of Australia are also included into *Permopsyche*: *Permopsyche belmontensis* (Riek, 1953), **comb. n.**, *Permopsyche robustus* (Riek, 1953) **comb. n.** The first pre-Triassic *Mesopsyche*, *Mesopsyche incompleta* **sp. n.** is described from the uppermost Permian (the town of Vyazniki, Vladimir Province). *Bittacopanorpa javorskii* Zalessky, 1935 from the uppermost Permian or basal Triassic of Kuznetsk Basin is identified as a hindwing of *Mesopsyche*: *Mesopsyche javorskii* (Zalessky, 1935) **comb. n.** The origin, evolutionary history, and stratigraphic occurrence of Mesopsychidae are discussed.

## Introduction

**Figure 1.**
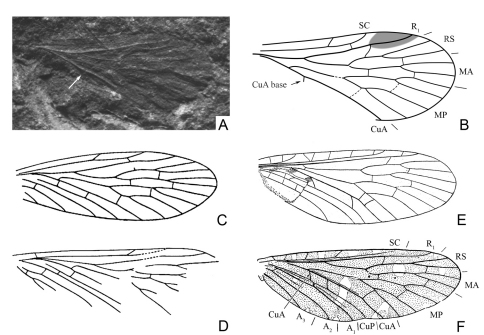
Wings of *Mesopanorpodes* and *Mesopsyche*, for comparison **A** photograph of *Mesopanorpodes wianamattensis* Tillyard (position of CuA base marked with arrow) **B** *Mesopanorpodes wianamattensis*, line drawing (orig.) **C** *Mesopanorpodes belmontensis*: from Riek (1953) **D** *Mesopanorpodes robustus*:from Riek (1953) **E** *Mesopsyche triareolata* Tillyard (= *Aristopsyche superba* Tillyard): from Tillyard, 1919a **F** *Mesopsyche dobrokhotovae* Novokshonov, modified after Novokshonov (1997c). Not to scale.

Tillyard (1917b) described *Mesopanorpa wianamattensis* from the Middle Triassic Ashfield Formation of the Wianamatta Group in New South Wales as an aberrant Mecoptera and erected the family Mesopanorpidae. Later (Tillyard 1918) he changed the genus name to *Mesopanorpodes* to avoid homonymy with *Mesopanorpa* Handlirsch, 1906. Subsequently (Tillyard 1919b: 611–612) he decided that *Mesopanorpodes* was closely related to *Mesopsyche* and the allied genera *Aristopsyche*, *Triassopsyche*, and *Neuropsyche* (the three latter have eventually been synonymized under *Mesopsyche* by Riek 1956), all described from the Upper Triassic Blackstone Formation, Queensland (Tillyard 1917a; Fig. 1E), and transferred *Mesopanorpodes* into the order Paratrichoptera.

Confusingly, this remark passed completely unnoticed by all the subsequent authors who discussed the composition and taxonomy of the Mesopanorpodidae. Riek (1953) placed into Mesopanorpodidae two new species of *Mesopanorpodes*, *Mesopanorpodes belmontensis* and *Mesopanorpodes robustus* (Fig. 1C–D), and the genus *Prochoristella* Riek, 1953 with six species from the Upper Permian of New South Wales (Belmont fossil beds). Since then, *Mesopanorpodes* and *Prochoristella* have been considered together either as forming a separate family Mesopanorpodidae (Riek 1955, 1974, Jell and Duncan 1986, Willmann 1978, 1989, Carpenter 1992, Van Dijk and Geertsema 1999, Hong et al. 2002, Hong and Guo 2003, Hong 2007, Sun et al. 2007a, b), or as two closely related genera within Permochoristidae (Martynova 1962, Novokshonov 1997b, Novokshonov et al. 2004).

In fact, species of *Prochoristella* (with the possible exception of *Prochoristella belli* Riek, 1953, known only from a hindwing) display characters typical of Permochoristidae, that is, first of all, a deep fork on MA vein and an oblique base of CuA, either fused with MP for a distance, or forming cubito-median Y-vein. *Prochoristella* and *Mesopanorpodes* are similar only in the number of RS+MA and MP endings being reduced to four. However, this is characteristic of many independent lineages, e.g. Permotanyderidae + Liassophilidae, Nannochoristidae, or Bittacidae, and cannot be accepted as a reliable apomorphy. I consider *Prochoristella*, including its possible junior synonyms (or at least closely related genera) *Afristella* Riek, 1974, *Mesotanyderus* Riek, 1955, and *Austrochoristella* Willmann, 1989, as belonging to Permochoristinae (Permochoristidae) and not related to *Mesopanorpodes*.

Equally unacceptable is the assignment to Mesopanorpodidae of a number of species from the mid-Triassic Tongchuan Fm of Shaanxi Province, China, described by Hong You-Chong and co-authors (Hong et al. 2002, Hong and Guo 2003, Hong 2007). *Erdosia pectinata* Hong, Guo & Wang, 2002 can be placed with certainty into Parachoristidae, as indicated by the typical pectinate RS and 5- or 6-branched MP. *“Mesopanorpodes” shaanxiensis* Hong, Guo & Wang, 2002 is based on a highly incomplete hindwing and may belong to either Parachoristidae, Permochoristidae, or Mesopsychidae. The systematic position of *Ladinochorista lata* Hong, 2007 is uncertain because of the poor preservation of the holotype. Several other taxa described in Mesopanorpodidae do not even belong to Mecoptera. *Triassochoristites jinsuoguanensis* Hong & Guo 2003 has already been recognized as a member of the dipteran family Vladipteridae (Blagoderov et al. 2007). *Forcinerva tongchuanensis* Hong and Guo 2003 belongs to the order Miomoptera (as determined by A.P. Rasnitsyn). *Allochorista erdosensis* Hong, 2007 and *Longifurcula hejiafangensis* Hong, 2007 cannot be attributed to any particular order based on the published photos and drawings. It should be noted that many descriptions of fossil insects from the Tongchuan Formation, and particularly Mecoptera, are based on inadequately preserved specimens and contain inaccurate drawings and fantastic venational interpretations, in which obvious artifacts are interpreted as characters of specific or even generic rank and hindwings are often misinterpreted as forewings. Thus, these taxa should be considered as of unknown systematic position until revised in detail.

The Jurassic genera *Itaphlebia* Sukatcheva, 1985 and *Chrysopanorpa* Ren, 1995 were originally placed in Mesopanorpodidae as well. Novokshonov (1997a, b) synonymized these two genera and transferred them to Nannochoristidae. *Itaphlebia* appears to be the most common and widespread nannochoristid during the Jurassic. Its diagnosis, composition, and distribution, as well as the overall composition of the Mesozoic Nannochoristidae have been discussed recently by Liu et al. (2010).

Hong (2007) referred *Itaphlebia* again to Mesopanorpodidae and described *Itaphlebia tongchuanensis* from the Tongchuan Formation of Shaanxi. However, this species is not related to *Itaphlebia*, being instead rather similar to *Mesopsyche*, where it apparently belongs.

*Stylopanorpodes eurypterus*, *Netropanorpodes decorosus*,and *Netropanorpodes sentosus* Sun, Ren & Shih, 2007 (Sun et al. 2007b) from the mid-Jurassic Jiulongshan Formation of Inner Mongolia, China, originally placed in Mesopanorpodidae, are venationally similar to the species of *Itaphlebia* occurring in the same deposits (Sun et al. 2007c, Liu et al. 2010) and belong to Nannochoristidae as well.

*Mesopanorpodes mostovskii* Novokshonov et al., 2004 from the basal Triassic of Northern Russia has been transferred to the related family Nedubroviidae (Bashkuev 2011).

Therefore, with the above-mentioned taxa discarded, Mesopanorpodidae remain composed of only three species of *Mesopanorpodes* from the Upper Permian and Middle Triassic of Australia. But are they actually congeneric?

Re-examination of available photographs of *Mesopanorpodes wianamattensis* reveals that its CuA is distinctly bent distal to the origin of MP, and the base of CuA is shifted distad and inclined backwards, like in *Mesopsyche* (Fig. 1A–B). Thus, *Mesopanorpodes* appears venationally indistinguishable from *Mesopsyche*. Here I provisionally retain *Mesopanorpodes* as a separate genus until the complete revision of *Mesopsyche*. Unlike *Mesopsyche wianamattensis* and *Mesopsyche*, both species described by Riek have the base of CuA short and transverse, with the intercubital space not expanded, which is presumed to be a plesiomorphic condition. These species are included here in the new genus *Permopsyche* gen. n.

In this paper, two new species of *Permopsyche*, *Permopsyche issadensis* sp. n. (type species)and *Permopsyche rasnitsyni* sp. n., are described from the Upper Permian of European Russia. These are the oldest currently known records of Mesopsychidae. The new material includes a number of well-preserved fore- and hindwings, which allow establishing yet another difference between *Permopsyche* and *Mesopsyche*, the position of the hindwing CuA vein with respect to the membrane. In *Permopsyche issadensis* CuA is concave (while the R1 is convex), as is typical of Permochoristidae, while in *Mesopsyche* spp., including the Permian species, as well as the other Mesozoic mesopsychid genera, both CuA and R1 are convex, like in most post-Paleozoic Mecoptera.

The earliest record of *Mesopsyche*, and the only one known so far to be definitely pre-Triassic, *Mesopsyche incompleta* sp. n., is described from the uppermost Permian of the European Russia.

*Bittacopanorpa javorskii* Zalessky, 1936 from the transitional Permian–Triassic sequence of the Kuznetsk Basin (Babiy Kamen’ locality, Maltsevo Formation), known only from a wing base (Fig. 8G–H), originally placed in Neorthophlebiidae and tentatively transferred to Permotipulidae (Bechly and Schweigert 2000), can be identified as a hindwing with venation almost identical to that of the above-mentioned *Mesopsyche incompleta* sp. n. (Fig. 8B).

## Material and methods

The study is based on examination of ca. 60 fossil specimens from the collection of Borissiak Paleontological Institute, Russian Academy of Sciences, Moscow (PIN). The holotype of *Bittacopanorpa javorskii* Zalessky is preserved in the collection of the Central Research Geological Prospecting Museum, Saint Petersburg (CRGPM).

Fifty-three isolated wings of *Permopsyche* were collected from a lens of fluvio-lacustrine deposits within the Sukhona River section, in Vologda Province, north-central Russia (Isady locality). It corresponds to the Kalikino Member of the Poldarsa Formation, and is dated the latest Severodvinian (correlated with the Lower Wuchiapingian: Golubev in press).

Five specimens of *Mesopsyche* were collected from the Balymotikha locality, an outcrop of lacustrine deposits of Vyazniki Permian–Triassic sequence, at the town of Vyazniki, Vladimir Province, central Russia. It is dated the latest Vyatkian (Late Changhsingian), somewhat below the Permian–Triassic boundary (Newell et al. 2010).

The fossils were examined with a Leica M165C stereomicroscope and photographed using an attached Leica DFC 425 digital camera. Images were digitally processed with Helicon Focus v. 5.1 and Adobe Photoshop CS3 graphic software. Line drawings were made using Inkscape v. 0.48 vector graphics editor.

On the line drawings the wings are oriented with their apices to the right, while some photographs are reversed left-right for better comparison.

The wing venation terminology follows Novokshonov (2002).

## Systematic paleontology

### Genus Mesopsychidae

Tillyard, 1917

http://species-id.net/wiki/Mesopsychidae

Mesopanorpodidae Tillyard, 1918, syn. n.

#### Included genera.

*Mesopsyche* Tillyard, 1917, *Mesopanorpodes* Tillyard, 1918, *Vitimopsyche* Novokshonov & Sukatsheva, 2001, *Baissopsyche* Novokshonov & Sukatsheva, 2001, *Lychnomesopsyche* Ren et al., 2009, *Permopsyche* Bashkuev, gen. n.

#### Remarks.

 The genus *Mesopanorpodes* is restricted here to its type species, *Mesopanorpodes wianamattensis*. Though *Mesopanorpodes* appears venationally indistinguishable from *Mesopsyche*, it doesn’t seem appropriate to synonymize these genera prior to the complete revision of the latter. The genus *Mesopsyche* (*sensu* Novokshonov 1997c, Novokshonov and Sukacheva 2001) includes apparently quite diverse insects and is quite loosely defined and likely heterogeneous, so its further dividing into several genera cannot be excluded.

### Genus Permopsyche gen. n.

urn:lsid:zoobank.org:act:57B809F6-634A-446C-AB88-79BE4E3E745C

http://species-id.net/wiki/Permopsyche

Mesopanorpodes : Riek, 1953, p. 70 (partim, quoad *Mesopanorpodes belmontensis* and *Mesopanorpodes robustus*).

#### Type species.

*Permopsyche issadensis* sp. n.; Upper Permian, European Russia.

#### Etymology.

 From the Permian and Greek *psyche*, “soul” or “mind,” the word often used for devising names of delicately winged insects. Gender feminine.

#### Diagnosis.

 In forewing, SC long, bearing only inclined fore branch, connecting distally with R1 by crossvein. Costal space narrow. Both RS and MA forks not longer than their stems. MP 4-branched. CuA base oblique to transverse, not distinctly inclined backwards; M5 present or lost. Anal area not expanded. Crossveins not numerous. In hindwing, CuP concave with respect to membrane (in contrast to convex R1).

#### Comparison.

 Differs from the closest genus *Mesopsyche* by the base of CuA being oblique to transverse (not inclined backwards), costal space narrower, and hindwing CuP concave with respect to membrane.

#### Composition.

 In addition to the type species, from the same locality, *Permopsyche rasnitsyni* sp. n.;and from the Upper Permian of Australia, *Permopsyche belmontensis* (Riek, 1953) comb. n. and *Permopsyche robustus* (Riek, 1953) comb. n.

#### Remarks.

 The assumption that one of the key characters, the position of hindwing CuP with respect to the membrane, known in the type species only, is shared by the other species is tentative and requires further verification.

### Permopsyche issadensis sp. n.

urn:lsid:zoobank.org:act:FBCADCA5-E2B6-4D4E-B044-6DE29496AAFD

http://species-id.net/wiki/Permopsyche_issadensis

Figs 2 [Fig F3] 4 6

#### Etymology.

 From the Isady locality.

#### Holotype.

 PIN, no. 3840/336, well-preserved forewing (part and counterpart).

#### Paratypes.

 21 forewings (see Table 1) and 6 hindwings: PIN, no. 3840/725, 1381–1383, 1385, 1389a. Additionally, 8 forewings and 10 hindwings incomplete or poorly preserved, excluded from the type series.

#### Locality and horizon.

 Isady locality, Vologda Province, North European Russia; Poldarsa Formation, uppermost Severodvinian (Lower Wuchiapingian), Upper Permian.

#### Diagnosis.

Differs from *Permopsyche belmontensis* by wing shape, with anterior margin straight and posterior apical margin tending to oblique, different crossveins arrangement, and strongly unsclerotized MP stem. Differs from *Permopsyche robustus* by branch of SC located more basally and A3 simple. Additionally differs from both species by considerably smaller size.

#### Description.

 Forewing. Moderately broad (length/width ratio 2.3–2.9:1); anterior margin nearly straight, slightly convex basally; apex obtuse to rounded. SC long, reaching or almost reaching basal margin of pterostigma; SC branch approximately at middle of RS+MA stem (somewhat variable in paratypes). Pterostigma distinct, widened, with oblique basal margin. R1 simple, gently bending posteriad at pterostigma. RS and MA forks equally short, approximately as long as their stems. Nodal line distinct, arched, running from tip of SC to hind margin at apex of CuA. Thyridium present as unsclerotized section on MP stem spanning from approximately 1/3–1/2 of its length down to fork, accompanied by desclerotized spot at point of branching of RS and MA (“thyridulum”, the term introduced by A.P. Rasnitsyn in Ren et al. 2009). Connection between CuA and MP variable (Fig. 4; see also Remarks), from joined at one point (X-junction), with CuA base oblique and M5 lost, to forming well-developed cubito-median Y-vein with almost equal arms at obtuse angle, with CuA base transverse and M5 quite long. cua-cup crossvein transverse. Three simple anal veins. Crossveins not numerous, mainly distinct or slightly weakened, forming rather stable pattern: one long, oblique, sigmoidally curved r1-rs crossvein; four crossveins between RS, MA, and MP branches; mp-cua and cua-cup crossveins of standard position; cup-a1 and a1-a2 located stepwise along with the base of CuA; occasionally, a2-a3 present. Wing membrane darkened in distal wing half, restricted by nodal line, with saturated patch at center of wing.

**Figure 2.**
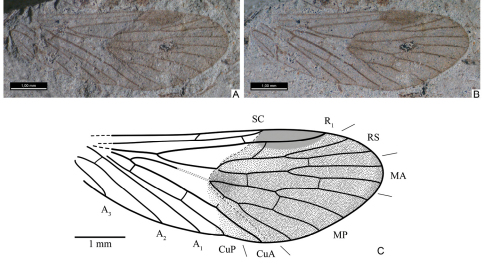
*Permopsyche issadensis* gen. et sp. n. **A–B** photographs of the holotype, under different illumination **C** line drawing of holotype.

**Figure 3. F3:**
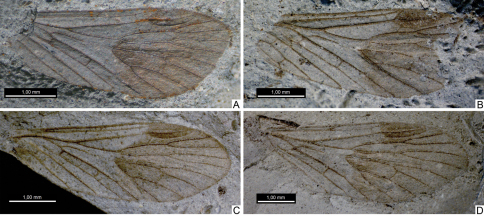
Photographs of forewings of *Permopsyche issadensis* gen. et sp. n. **A** paratype PIN, no. 3840/717; **B** paratype PIN, no. 3840/1354 **C** paratype PIN, no. 3840/1355 **D** paratype PIN, no. 3840/1357. All except **D** reversed left-right.

**Figure 4.**
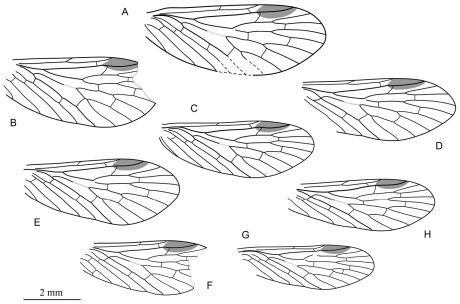
Line drawings of paratypes of *Permopsyche issadensis* gen. et sp. n. showing the variability of venation and wing size **A** PIN, no. 3840/718 **B** PIN, no. 3840/719 **C** PIN, no. 3840/105 **D** PIN, no. 3840/1357 **E** PIN, no. 3840/1352 **F** PIN, no. 3840/1354 **G** PIN, no. 3840/728 **H** PIN, no. 3840/729. All to the same scale.

Hindwing. Both venation scheme and coloration similar to those in forewing, adjusted for general difference between fore- and hindwings typical of Mecoptera. SC short, reaching wing midlength, variably forking apically (Fig. 6). Pterostigma distinct. R1 with inclined branch in pterostigmal area. A1 separating from CuP relatively closely to base.

**Figure 5.**
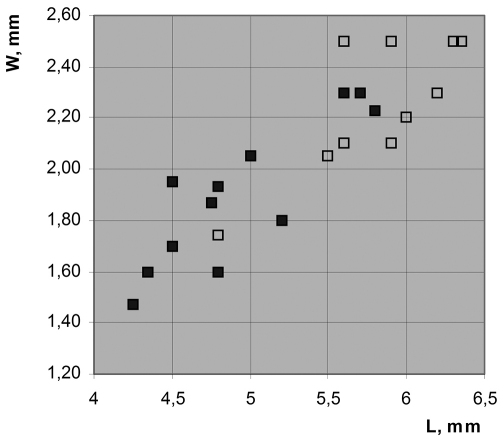
Correlation between wing size and structure of connection between CuA and MP in *Permopsyche issadensis*, based on Table 1; see text for explanation.

**Figure:**
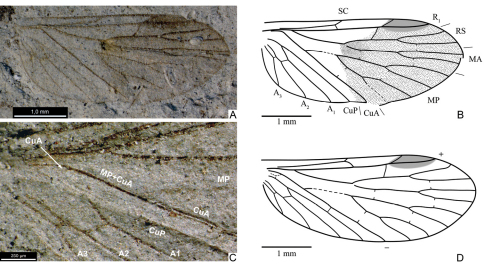
**Figure 6.** Hindwings of*Permopsyche issadensis* **gen. et** **sp. n.** **A–B** paratype PIN, no. 3840/1381, photograph and line drawing **C–D** paratype PIN, no. 3840/1382 **C** details of cubito-anal structure **D** line drawing of hindwing **A,** **C** reversed left-right.

#### Measurements (mm).

 Forewing length, 4.25–6.35, width, ca. 1.5–2.5; hindwing length, 3.9–5.5, width, 1.5–2.25.

#### Remarks and discussion.

In *Permopsyche issadensis* thewing size varies conspicuously. As shown in the size distribution diagram for forewings (Fig. 5; Table 1), the presence of Y-vein and the transverse (rather than oblique) shape of CuA base (hollow squares *vs* filled squares) both correlate with the increased wing size (irrespectively of the length/width ratio). However, the size distributions of the two morphotypes overlap broadly. The two wing morphotypes may represent sexual dimorphism. Alternatively, they can be attributed to slight differences in flight mechanics depending on the body size. The distinctive and uniform wing color pattern makes it unlikely that the morphotypes represent two closely related species.

**Table 1.** Forewing sizes of *Permopsyche issadensis* gen. et sp. n. Some measurements are acceptably approximate due to restoration of wing base and/or apex.

*PIN, no. 3840/…*	*Length*	*Width*	*L:W*	*CuA/MP fusion*
730	4.25	1.47	2.89	■
1362	4.35	1.6	2.72	■
1360	4.5	1.7	2.65	■
723	4.5	1.95	2.31	■
1367	4.75	1.87	2.54	■
728	4.8	1.6	3.00	■
1355	4.8	1.74	2.76	□
1354	4.8	1.93	2.49	■
1359	5.0	2.05	2.44	■
729	5.2	1.8	2.89	■
105	5.5	2.05	2.68	□
1349	5.6	2.1	2.67	□
1352	5.6	2.3	2.43	■
719	5.6	2.5	2.24	□
1358	5.7	2.3	2.48	■
1357	5.8	2.23	2.60	■
1353	5.9	2.1	2.81	□
1350	5.9	2.5	2.36	□
1366	6.0	2.2	2.73	□
*336*	*6.2*	*2.3*	*2.70*	□
717	6.3	2.5	2.52	□
718	6.35	2.5	2.54	□

The nodal line (the line of wing flexion) in the forewing of the new species is essentially similar to that of both *Mesopsyche* (Novokshonov 1997c) and the recent *Panorpa* (Ennos and Wooton 1989), suggesting a similarity between flight mechanics of Mesopsychidae and Panorpidae.

Hindwings of *Permopsyche* are almost as abundant at Isady as the forewings. All the hindwings examined, as far as their preservation allows to tell, have the set of characters (including coloration) diagnostic of the forewings of *Permopsyche issadensis* and can be confidently referred to the same species.

### Permopsyche rasnitsyni sp. n.

urn:lsid:zoobank.org:act:E74CC301-D5B9-4093-9194-BCE6B1C852D2

http://species-id.net/wiki/Permopsyche_rasnitsyni

Fig. 7A–D

#### Etymology.

 In honorof an outstanding paleoentomologist, Prof. Dr. Alexandr Rasnitsyn.

#### Holotype.

 PIN, no. 3840/1351, well-preserved forewing (part and counterpart), with apex missing.

#### Paratypes.

 PIN, no. 3840/733, 1363, 1371, incomplete forewings.

#### Locality and horizon.

 Isady locality, Vologda Province, North European Russia; Poldarsa Formation, uppermost Severodvinian (Lower Wuchiapingian), Upper Permian.

#### Diagnosis.

Similar to *Permopsyche issadensis* ingeneral venation scheme, but of larger size, with MA fork shortened, intercubital space widened, and wing membrane almost uncolored.

#### Description.

 Forewing length/width ratio about 2.8–3:1; anterior margin nearly straight; apex obtuse to rounded. Costal space very narrow along its entire length. MA fork distinctly shorter than RS fork. MP unsclerotized for more than 1/2 of its length. CuA base long, nearly transverse, M5 present or lost. Intercubital space rather expanded. Wing membrane mostly uncolored, with small diffuse dark patch at center of wing.

**Figure 7.**
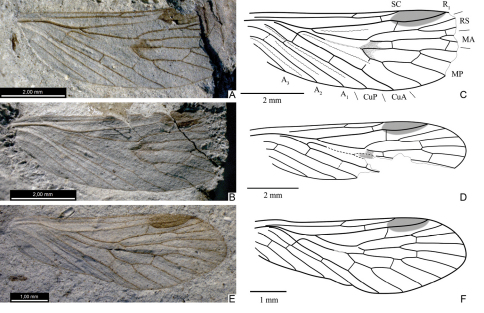
**A–D** forewings of *Permopsyche rasnitsyn*i gen. et sp. n. **A–B** photograph of holotype, PIN, no. 3840/1351, part and counterpart **C** line drawing of holotype, PIN, no. 3840/1351 **D** line drawing of paratype, PIN, no. 3840/1363 **E–F** *Permopsyche* sp., photograph and line drawing of specimen PIN, no. 3840/1556.

#### Measurements (mm).

 Holotype length, as preserved, 6.7, width, 2.5; paratypes lengths, ca. 7.0–8.5, width unmeasurable due to widthwise deformation.

### Permopsyche sp.

Fig. 7E–F

#### Material.

 PIN, no. 3840/1556, well-preserved forewing (part and counterpart).

The wing is 6 mm long, 2.1 mm wide, subtriangular, with anal area narrowed and posterior apical margin slightly oblique; MA forking before RS. The wing is entirely uncolored (neither part, nor counterpart showing any trace of coloration). The specimen may either be an aberrant specimen of *Permopsyche issadensis*, or represent a different species, but describing a separate species based on a single wing does not seem well justified.

### Genus Mesopsyche

Tillyard, 1917

http://species-id.net/wiki/Mesopsyche

Triassopsyche Tillyard, 1917: Riek 1956, p. 100.
Aristopsyche Tillyard, 1919: Riek 1956, p. 100.
Neuropsyche Tillyard, 1919: Riek 1956, p. 100.
Mesoses Riek, 1976: Novokshonov and Sukatcheva 2001, p. 71.
Bittacopanorpa Zalessky, 1935, syn. n.
Itaphlebia Hong, 2007, syn. n. (partim, quoad *Itaphlebia tongchuanensis*)

#### Type species.

*Mesopsyche triareolata* Tillyard, 1917; Upper Triassic, Australia.

#### Diagnosis.

 Forewing relatively broad. SC rather long, with single or multiple fore branches. Costal space typically expanded. Both RS and MA forks not longer than their stems. MP 4-branched. CuA base from transverse to distinctly inclined backwards, usually situated much more distally than MP origin. Crossvein cua-cup of same inclination as CuA base. Intercubital space rather expanded. Crossveins not numerous, their arrangement rather stable. In hindwing, CuP convex with respect to membrane; fused with A1 very close to base.

#### Comparison.

 Differs from *Permopsyche* by the base of CuA being inclined backwards, costal space widened, and hindwing CuP convex.

#### Composition.

 In addition to the type species, from the beds near the Permian–Triassic boundary of Kuznetsk Basin, *Mesopsyche javorskii* (Zalessky, 1935) comb. n.; from the Middle Triassic of China, *Mesopsyche tongchuanensis* (Hong, 2007) comb. n.; from the Middle–Upper Triassic Madygen Formation of Kyrgyzstan, *Mesopsyche shcherbakovi*, *Mesopsyche justa*, *Mesopsyche ordinata*, *Mesopsyche tortiva*, and *Mesopsyche gentica* (Novokshonov 1997c, Novokshonov and Sukatcheva 2001); from the Upper Triassic of South Africa, *Mesopsyche optata* (Riek, 1976), *Mesopsyche magna* (Riek, 1976); from the Upper Triassic of Ukraine, *Mesopsyche dobrokhotovae* (Novokshonov 1997c).

### Mesopsyche incompleta sp. n.

urn:lsid:zoobank.org:act:FC61FFCE-6E4D-401F-BD62-227A6E376D1B

http://species-id.net/wiki/Mesopsyche_incompleta

Fig. 8

#### Etymology.

 Latin *incompletus*, “incomplete”.

#### Holotype.

 PIN, no. 5103/286, hindwing (part and counterpart), with cubito-anal area missing.

#### Paratypes.

 PIN, no. 5103/122, incomplete forewing (basal half only), part and counterpart; PIN, no. 5103/123, incomplete forewing (anterior half only, with apex, basal and posterior parts missing), part and counterpart. In addition, two forewing fragments apparently belonging to the same species.

#### Locality and horizon.

Balymotikha locality, Vyazniki town, Vladimir Province, Central European Russia; uppermost Vyatkian (Upper Changhsingian), Upper Permian.

#### Description.

 Forewing. Anterior margin slightly convex. Costal space somewhat wider than subcostal one. Pterostigma distinct, lanceolate. SC not reaching the pterostigma, bearing one oblique distal branch; connected distally with R1 by short transverse crossvein. R1 sharply curved posteriad at origin of weak distal branch in pterostigmal area. RS forking somewhat before than MA. Thyridium at MP stem before fork. CuA base obscure, presumably curved backwards and located somewhat distal to M5. Crossvein cua-cup slightly sigmoid, distinctly curved backwards. Crossvein pattern typical of genus. Color pattern in form of sparse dark spots, mostly around crossveins.

Hindwing venation and coloration similar to those in forewing. SC slightly shortened, reaching level of RS+MA bifurcation, somewhat beyond wing midlength, forking apically. Pterostigma distinct. R1 nearly straight, turning apically towards anterior margin. R1 branch obscure. CuA distinctly convex along the whole length.

**Figure 8.**
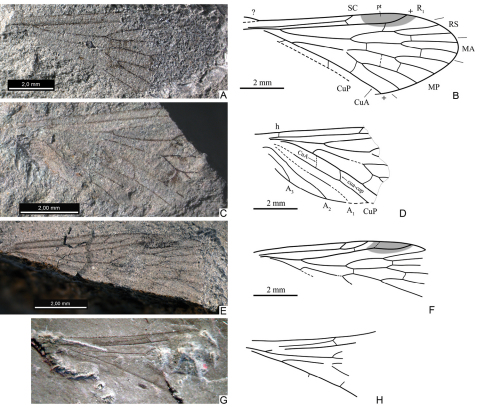
**A–F**, wings of *Mesopsyche incompleta* sp. n. **A–B** holotype PIN, no. 5103/286, hindwing, photograph and line drawing **C–D** paratype PIN, no. 5103/122, forewing, photograph and line drawing **E–F** paratype PIN, no. 5103/123, forewing, photograph and line drawing **G–H** *Mesopsyche javorskii* (Zalessky) comb. n., holotype hindwing, photograph (by D. Shcherbakov) and line drawing (orig.), not to scale **A,** **C,** **G** reversed left-right.

#### Measurements (mm).

 hindwing length of holotype, 9.8; forewing length of paratype PIN, no. 5103/122, 6.1, as preserved; paratype PIN, no. 5103/123, 7.9, as preserved.

#### Comparison.

 The new species substantially differs from others by SC with a single, distally located fore branch.

## Discussion

The systematic position of Mesopsychidae and the entire “suborder” Paratrichoptera was discussed by Novokshonov and Sukatcheva (2001). Recent studies of complete mesopsychid fossils from the Middle Jurassic and Lower Cretaceous of China revealed the long siphonate mouthparts indicating fluid feeding on reproductive organs of gymnosperm plants (Ren et al. 2009, 2010). Mesopsychidae, together with mid-Mesozoic Aneuretopsychidae, Pseudopolycentropodidae, and now the Late Permian to Early Triassic Nedubroviidae (Bashkuev 2011), form a distinct long-proboscid clade within Mecoptera, the Aneuretopsychina.

The finds described herein reveal main trends in the evolution of Mesopsychidae after their separation from the permochoristid-like ancestor. These are:

– Change in the forewing CuA base inclination and position: from proximal and inclined forwards (typical of Permochoristidae) to inclined backwards, and probably also shifted distad.

– Change in position of the hindwing CuA: from concave (*Permopsyche*) to convex (most Mesopsychidae).

– Loss of fusion of CuP and A1 veins in the hindwing: from quite basally fused in *Permopsyche*, through a rudimentary basal fusion in *Mesopsyche* (as indicated by Novokshonov (1997c) in *Mesopsyche shcherbakovi*), to becoming secondary free along their whole length in the mid-Jurassic and early Cretaceous genera.

– Increase in the average wing size, from 5–6 mm in the Late Permian to 20–30 mm since the Late Triassic.

The Permian Mesopsychidae occurred together with Nedubroviidae (in Isady, Balymotikha, and probably Belmont; Bashkuev 2011), which already have typical long siphonate mouthparts, and the possession of those in early mesopsychids (and even in the ancestral permochoristid lineage) can be assumed as well.

New finds also cast light on the stratigraphic distribution of the Aneuretopsychina scorpionflies, which appear to be the most common group of Mecoptera during the latest Permian – early Middle Triassic time interval (viz. Mesopsychidae, Nedubroviidae, and later also Pseudopolycentropodidae). They initially replaced Permochoristidae in the end of Permian, and were in turn progressively supplanted during the Triassic – Early Jurassic by Parachoristidae and Orthophlebiidae, which gave rise to the modern mecopteran lineages. In the mid-Jurassic – Lower Cretaceous insect assemblages, Aneuretopsychina scorpionflies are rather rarely found, constituting only a minor component of the mecopteran faunas.

## Supplementary Material

XML Treatment for Mesopsychidae

XML Treatment for Permopsyche

XML Treatment for Permopsyche issadensis

XML Treatment for Permopsyche rasnitsyni

XML Treatment for Permopsyche

XML Treatment for Mesopsyche

XML Treatment for Mesopsyche incompleta
